# General Practitioners—Public Dinner

**Published:** 1830-07-01

**Authors:** 


### XVII.

### GENERAL PRACTITIONERS.

The public dinner for promoting a measure or mode of practice which we have long recommended, is at length fixed; and, although we are convinced that the said measure *must* ultimately come into full operation, we are not without fears that the poisonous leaven of furious party-spirit, which has for some time been boiling in the cauldron of professional agitation and degradation, will diffuse, or, at all events, attempt to diffuse its baleful influence among an assemblage of practitioners, the great majority of whom are meeting for good and legitimate purposes. The eyes of their brethren are on their proceedings. And if they suffer themselves to be led away from the path of sober sense by the apple of discord, which will be artfully and furiously launched forth, after the Tuscan grape has excited the feelings at the expense of the judgment, the loss will be theirs, though the fate of this warning be that of Cassandra's prophecies.

